# ‘Doing more with less’: a qualitative investigation of perceptions of South African health service managers on implementation of health innovations

**DOI:** 10.1093/heapol/czz017

**Published:** 2019-03-12

**Authors:** Carrie Brooke-Sumner, Petal Petersen-Williams, James Kruger, Hassan Mahomed, Bronwyn Myers

**Affiliations:** 1 Alcohol, Tobacco and Other Drug Research Unit, South African Medical Research Council, Francie Van Zijl Drive, Parow Valley, Cape Town, South Africa; 2 Department of Psychiatry and Mental Health, University of Cape Town, J-Block, Groote Schuur Hospital, Observatory, Cape Town, South Africa; 3 Western Cape Government: Health, Norton Rose House, 8 Riebeeck Street, Cape Town, South Africa and; 4 Division of Health Systems and Public Health, Department of Global Health, Faculty of Health Sciences, Stellenbosch University, Francie van Zijl Drive, Tygerberg, Cape Town, South Africa

**Keywords:** Primary care, innovation adoption, managers, resilience, mental health

## Abstract

Building resilience in health systems is an imperative for low- and middle- income countries. Health service managers’ ability to implement health innovations may be a key aspect of resilience in primary healthcare facilities, promoting adaptability and functionality. This study investigated health service managers’ perceptions and experiences of adopting health innovations. We aimed to identify perceptions of constraints to adoption and emergent behaviours in response to these constraints. A convenience sample of 34 facility, clinical service and sub-district level managers was invited to participate. Six did not respond and were not contactable. In-depth individual interviews in a private space at participants’ place of work were conducted with 28 participants. Interviews were audio recorded and transcribed verbatim. NVivo 11 was used to store data and facilitate framework analysis. Study participants described constraints to innovation adoption including: staff lack of understanding of potential benefits; staff personalities, attitudes and behaviours which lead to resistance to change; high workload related to resource constraints and frequent policy changes inducing resistance to change; and suboptimal communication through health system structures. Managers reported employing various strategies to mitigate these constraints. These comprised (1) technical skills including participatory management skills, communication skills, community engagement skills and programme monitoring and evaluation skills, and (2) non-technical skills including role modelling positive attitudes, understanding staff personalities, influencing perceptions of innovations, influencing organizational climate and building trusting relationships. Managers have a vital role in the embedding of service innovations into routine practice. We present a framework of technical and non-technical skills that managers need to facilitate the adoption of health innovations. Future efforts to build managers’ capacity to implement health innovations should target these competencies.

**Key Messages**

- This study investigated health service managers’ perceptions and experiences of adopting health innovations.
- Constraints to innovation adoption identified were: staff lack of understanding of potential benefits; staff personalities, attitudes and behaviours; high workload related to resource constraints and frequent policy changes.
- Managers navigated these challenges using participatory management skills, communication skills, community engagement skills, and non-technical skills including role modelling, staff personalities, influencing perceptions and organizational climate and building trusting relationships.
- Future efforts to build managers capacity to implement health innovations should target these competencies

## Introduction

Low- and middle-income countries (LMICs) face a complex burden of disease, with the impact of chronic diseases mounting. The South African public health system is vulnerable to this intensifying burden, which includes mental, neurological and substance use disorders and their multi-morbidity with chronic communicable and non-communicable diseases (Mayosi *et al.*, 2012). The economic climate presents fiscal constraints that challenge health system responsiveness to this burden. The South African Mental Health Policy and Strategic Framework (2013–2020) has promoted non-specialist delivery of manualized psychosocial support services in the chronic disease care platform (DoH, 2013; Mahomed *et al.*, 2014). Several studies are evaluating this approach in different provinces (Fairall *et al.*, 2018; Myers *et al.*, 2018; Petersen *et al.*, 2018); however, implementation of this innovation is complex. There are ongoing calls for building resilience in the health system overall (Elloker *et al.*, 2012–2013; Gilson *et al.*, 2017) with ‘software’ (e.g. management skills, relationships) being as important as hardware (e.g. drugs, instruments; Barasa *et al.*, 2017) in working towards this goal. Health service managers’ capability for implementing service changes based on policy goals may be a key aspect of system resilience through promoting adaptability, responsiveness and functionality (Barasa *et al.*, 2017) of primary healthcare (PHC) facilities.

Application of change management theory to healthcare organizations outlines the importance of organizational readiness for change (ORC; Weiner, 2009) and attitudes to evidence-based practices (Aarons *et al.*, 2011) in influencing adoption of innovations. ORC comprises an organization’s staff’s joint commitment to implement a change, and staff members’ shared belief in their capability to achieve the change (Weiner, 2009). A broad range of factors contribute to ORC, including organizational climate, staff’s openness to change, and staff relationships and competencies (Cresswell and Sheikh, 2013; Williams *et al.*, 2015). In the South African PHC context, staff and other resource shortages constrain ORC. In addition, teams of staff working in PHC can be viewed as a complex adaptive system, in which the outcomes achieved are based on interactions between team members (as components of the system) and ‘emergent behaviours’ based on these interactions (Sweeney, 2002). Such behaviours emerge as a fitting response in challenging environments in which innovative thinking and flexibility in testing of new approaches is the best way (Pype *et al.*, 2018) to address multi-level constraints (Aarons *et al.*, 2011). When given the flexibility to function in this way, a complex adaptive system can produce new strategies for achieving desired outcomes (Ellis and Herbert, 2011; Paina and Peters, 2012). Wider political and social contexts also influence health facilities as complex adaptive systems (Ellis and Herbert, 2011; Erasmus *et al.*, 2014). As custodians of this context, health service managers must rapidly adapt to changing policy directives and build relationships and staff resilience to weather challenges from within the health system and the communities they serve (Nyikuri *et al.*, 2015). In this top-down hierarchy, these managers also have a key role in determining how various tasks are prioritized, and their perceptions of policies and innovations exert a strong influence on implementation (Uvhagen *et al.*, 2018).

In South Africa, as in many other LMICs, PHC facilities are managed by professional nurses who are promoted to the role of facility or operational manager, often within the same facility, such that they may manage former peers. Some larger facilities have both a facility and operational manager, with the former focusing more on administrative tasks, but in most facilities the role is combined. These managers are experienced clinicians but have little training or mentoring to enable their development as managers (Daire and Gilson, 2014). They are expected to manage facility resources, shape the organizational culture, influence individuals and relationships, motivate all cadres of staff, sustain links with the various tiers of the health system and lead organizational change (Cook *et al.*, 2012; Daire and Gilson, 2014; Gilson, 2016). This study investigated health service managers’ perceptions and experiences of adopting service changes. We aimed to identify perceptions of constraints to innovation and emergent behaviours in response to these constraints. We planned to use these findings to inform the development of a framework of skills required for health managers to implement health innovations. Building managers’ capabilities for guiding the change process may help build health system resilience.

## Methods

This study was nested in the Project MIND cluster randomized controlled trial, which is evaluating two approaches to integrating counselling for depression and hazardous alcohol use into chronic disease care for HIV and diabetes patients. The MIND intervention is a three-session blended problem-solving-motivational interviewing intervention delivered by trained lay counsellors within primary health centres. This type of counselling is currently not available in clinics in the study area. Each session is ∼60 min long, and telephone booster sessions are offered 8 weeks after enrolment in the study (Myers *et al.*, 2018). The trial is being implemented in 24 PHC facilities in the Western Cape (16 intervention sites, 8 treatments as usual sites). This qualitative sub-study is presented according to COREQ guidance for reporting qualitative research (Tong *et al.*, 2007). A convenience sample of 34 facility, clinical service and sub-district level managers were contacted by email with an interview request. These were managers who had been involved in Project MIND and had recent experience of introducing a complex intervention to facilities. In cases where a facility and operational manager were present, both were contacted. Follow-up telephone calls were made. Six facility managers did not respond and were not contactable. These managers were from a variety of clinics, suggesting that lack of response was due to time constraints, or issues with email and telephone communication, which are known to occur in health facilities. This therefore should not have introduced a systematic bias to responses. The first author, an experienced, female qualitative researcher, with a background in mental health intervention development, conducted in-depth individual interviews in a private space at participants’ place of work. The researcher was a postdoctoral fellow, independent from Departmental of Health structures, working on the organizational aspects of introducing the MIND innovation. She was not involved in management or reporting relating to the trial, which may have reduced social desirability in responses. She was known to managers through interaction at a workshop introducing the study, but had not worked directly with managers. Written informed consent was obtained prior to the interview. The interview schedule employed a phenomenological approach aiming to gain a rich description of lived contexts (Davidsen, 2013) and experiences of introducing new programmes or services. Participants were asked about their role and experiences in introducing new programmes or services into their facilities, the process of innovation adoption within the health system, and their opinions on constraints and facilitators in their context (see Supplementary Appendix 1). Interviews were conducted in English and lasted between 45 and 60 min. English is the official business language used in the South African health system. Managers are accustomed to using English in workplace communication and conducting interviews in English was not expected to affect responses. Two managers responded in a mixture of English and Afrikaans, which is common in verbal communication in the Western Cape province. Interviews were transcribed verbatim. Afrikaans sections were transcribed and translated by a bilingual member of the research team. NVivo 11 was used to store data and facilitate analysis using the framework approach (Gale *et al.*, 2013). This approach combined inductive and deductive coding. It captured specific themes while leaving flexibility for new themes to emerge particularly in relation to minority perspectives, and previous experiences of managers. The first author conducted the initial process of familiarization with the data through review, initial coding and identification of major themes. The first and second author independently coded the first five transcripts and then met to refine codes and themes. Coding then continued independently until saturation of data. Any coding disagreements were resolved through consultation with the third author.

## Results

Sixteen facility managers, four clinicians involved in service management, and eight sub-district level managers from facilities participating in MIND were interviewed. Most participants were female (five male) consistent with the predominantly female staffing profile of PHC facilities in South Africa. The majority of participants was from Project MIND intervention sites, and described their experience in relation to the resource and service organization for implementing the Project MIND intervention. A subgroup of participants drew out experiences relating to implementation of the IDEAL clinic programme. This is a national quality improvement programme introduced in 2015 in which primary care facilities complete a yearly audit against requirements for: adequate infrastructure, adequate staff, adequate medicines and supplies, good administrative processes, applicable clinical policies, protocols and guidelines (Hunter *et al.*, 2017). The remaining minority of participants related their experience in relation to specific Department of Health policy implementation [e.g. changes to protocol for prevention of mother to child transmission of HIV (PMTCT0]). Analysis revealed several emergent behaviours in response to identified constraints to innovation adoption (see Table 1). These findings may be relevant to introduction of complex innovations such as the MIND intervention, as well as a variety of service delivery changes aligned with the IDEAL clinic programme.

**Table 1:** Constraints and emergent behaviours of health service managers supporting health innovation adoption

Constraints to innovation adoption	Emergent behaviours and capabilities —managers’ responses to constraints
Individual staff constraints	

Staff’s lack of understanding of the innovation and its potential benefits	Influencing staff’s perception on the value of the innovation Communicating public health perspectives and the value of a specific innovation Communicating links between innovations and Department of Health goals
Staff personalities, attitudes and behaviours lead to resistance to change	Understanding staff’s personalities, motivations, skills and interests Working with gatekeepers to influence perceptions and practices Role modelling positive attitude to change and commitment to the community Employing strategies to motivate staff Gaining community perspectives on innovation and communicating these with staff

Health system constraints	

Workload inducing resistance to change Suboptimal communication through DOH structures Hierarchy of health system limits managers decision-making innovations Frequency and pace of change is overwhelming	Communicating the ‘bigger picture’ of reduced patient visits (benefit to facility and staff) Working with staff unions to influence perceptions of innovation Responding quickly to staff concerns Providing positive feedback and regular communication about innovation adoption Facilitating teambuilding Finding non-financial ways to reward staff Rapidly internalizing and packaging innovation information for presentation to frontline staff Employing appropriate planning strategies to facilitate consultation with staff and implementation Using a participatory management style Adopting a collaborative planning approach to strengthen staff support while following policy directives. Executing a review mechanism for implementation of innovations to ensure sustained support Harnessing appropriate planning skills to drive the consultative planning process, piloting, engagement, response and feedback

### Constraints to innovation adoption

Constraints to innovation adoption were commonly described by facility, sub-district and clinical managers in themes outlined below. Participants were not asked specifically whether they experienced differences in constraints experienced depending on the initiator of the innovation (e.g. Department of Health (DoH) vs an ‘outside’ research organization). However, two participants noted that their experiences were similar whether implementing a DoH initiative, or a research study from an ‘outside’ institution. Managers reported that frontline staff largely viewed innovations as additional work imposed by higher structures and that this perception led to a lack of commitment to implementation of changes. This was commonly linked to the paucity of public health training among nurses and their limited understanding of the value of health innovations.

*Everybody is focused on their output and I think what we need to do is give them a bigger insight into a public health output, and actually if you spend more time doing the community work you should have less burden in your facility. But it’s historical. It’s how people have been trained. They’ve been trained in the bio-medical model and they want to fix people* (Participant 17).

Most managers expressed how personalities, attitudes and behaviours of staff reflected resistance to change. They related this to their high workload and uncertainty around new practices. Some managers acknowledged that certain staff accept new programmes and service changes more readily than others. They commented that younger staff were generally more accepting of innovations than older staff approaching retirement who were less open to change.

*I’ve noticed the younger* generation*…the newly appointed ones, they are easily influenced or motivated on something that is new. And they are so willing to change… so for now I think I'm still lucky that I'm appointing more young people that can understand where the department's [Department of Health] vision is going* (Participant 13).

According to participants, poor communication around the introduction of innovations contributes to resistance to change. Managers described challenges in communication from provincial, to district and sub-district tiers which impeded the flow of critical information to frontline staff. Specific challenges included: high frequency of communicating information via email ‘circulars’ yet frontline staff had limited access to email; poor attendance of frontline staff at meetings due to conflicting demands and workload; poor buy-in of staff excluded from initial communication about the innovation; and rapid introduction of the innovation to the facility with insufficient time for frontline staff to understand it before implementation.

*What I find is that sometimes the managers go to meetings and the managers get the protocols and all of that and they say ‘Yes, yes, it will benefit our facility.’ But then the people [staff] actually don’t know about it…and they just see it as extra work and they don’t do it* (Participant 27).

Constraints to innovation adoption at the health system level were frequently described. Insufficient staff to meet the growing patient burden was reported. Several managers described how this increased staff’s workload and made providing quality care difficult. These challenges were present in large and small facilities, and across rural and urban settings. According to participants, this increased workload without corresponding increased staffing contributed to high levels of absenteeism and low morale and was the fundamental reason for resistance to change. In contrast, the minority of managers who perceived their staff complement to be adequate thought it was feasible to implement health innovations with some adjustments to patient flow and staff tasks.

*Because we have a shortage of* staff *it's not always easy to implement something new…because we are under a lot of pressure, seeing a lot of people you tend to try to work faster but now you can imagine that one sister doing two people's work is not enough* (Participant 12).

The hierarchical culture of the health system was also a barrier to innovation adoption. Most managers described lacking agency and having limited involvement in decision-making. Instead, decisions around innovations are made by the upper tiers of the health system and channelled down to facility managers. Facility level managers referred to a lack of understanding from higher structures of the pressing challenges to service provision experienced by facilities. They described little opportunity for bottom-up communication, or consultation prior to the introduction of innovations. Their lack of influence over budgets, exclusion from meetings where decision-making on innovations occurs, and inadequate introduction to planned innovations further decreased their ownership of and investment in new health programmes. Sub-district level managers described their work to support facilities taking on new programmes or services, which they recognized as a key aspect of their role. They also highlighted the pressures exerted on them from both the higher and lower tiers of the health system.

*It’s just introduced and there’s no other follow up or support and that’s where things go wrong… they [subdistrict] should actually take the lead and actually assist with us, and not just be telling us what needs to be done, but be more actively involved with it* (Participant 16).

A further constraint was the pace and frequency of system changes. Managers at both the sub-district and facility level commonly described feeling overwhelmed by the number of changes they were required to implement, while acknowledging that these changes often brought about quality improvement. The timing of changes was perceived to be poorly managed, with many changes introduced over a short space of time. This demotivates staff and increases resistance to change. Several managers thought those in the higher tiers of the health system did not understand the burden and stress caused by constant change. Related to this, a subgroup of managers described the expectation on them to ‘do more with less’, adding new services or innovations without additional staff. They attributed this to a shrinking resource pool, flatlining of budgets and increasing patient numbers.

*Government have the best policies and* protocols*. But the sad thing is they add on to services but they don’t add on to staff. So, then it means that good policy and protocols it means nothing, because staff will take shortcuts* (Participant 22).

Some managers believed ‘doing more with less’ to be an unreasonable expectation which was stressful for managers and staff, created resistance to change, and reduced the quality of patient care.

*It is simply said there is a budget and this is the staff you are given, so you must just cope. Then they [substructure] say you must work ‘smarter’. I don’t know how to work smarter to get through more people* (Participant 26).

A subgroup of managers also described having limited time for management and administration tasks. This impacted on their capacity to promote the adoption of health innovations. In both rural and urban contexts, managers were frequently drawn into clinical work due to high patient burden. When this occurred, there was less time for management tasks. Participants recognized that their lack of time to guide and support staff in implementing new services impacted on the introduction of health innovations.

*So, those are the frustrations [time constraints] in the end…the support to the staff, once the programme or the change is introduced, that is the most important thing. It's like buying a car and* then *the car is broken within a month and no one wants to help you* (Participant 9).

Managers also noted that their lack of time impacted on their ability to develop as managers, which they felt affected their ability to support the adoption of health innovations. Although many acknowledged having access to a variety of training, coaching and mentorship opportunities, they reported difficulty in dedicating time for these activities. Only two participants had completed a full training or mentorship programme.

*It’s [*management *training] useful. Even myself, it’s not easy to take those eight sessions. I think I went for one, because now I want to go again, you know, but because of the time…there's no time* (Participant 11).

### Emergent behaviours: capabilities developed to support innovation adoption

Participants identified certain leadership characteristics as key to addressing individual staff constraints to innovation adoption**.** These included being a motivator, having a positive approach to challenges and leading by example (e.g. getting involved in clinical work where needed), showing passion for serving patients and their community and being proactive in addressing staff concerns. Managers who embodied these qualities viewed expectations to ‘do more with less’ as an opportunity to cultivate resilience among staff and improve services. These managers felt empowered in their management role and described strong agency in innovating for improving clinic functioning. Several described their role as one of ‘selling’ innovations to staff, which they recognized was dependent on their own understanding of and support for the particular innovation.

*If you’re not positive, then you can’t expect the rest of the staff to be positive and you won’t get anywhere with a negative approach. And like in the reception department, I’m on hand there, filing, drawing files…So I know the challenges that you’re battling with* (Participant 3).

Most managers underscored the need for staff to be motivated by understanding the ‘big picture’, i.e. not only the benefit of a change for their own work, but also how this change could improve population health.

*…you must help them to see that at the end it benefits the patient because they don't have to come to the clinic anymore, and that also helps less feet over the clinic's door… whereas if you maybe see something is not that effective and it doesn't help you or your patient a lot, you won't put in that much effort* (Participant 12).

Another strategy to address staff-related barriers to innovation adoption was to harness staff dedication and strengths. Through their experience of working closely with staff, managers identified individuals’ strengths, competencies and desire to acquire skills in specific interest areas. Managers worked through these staff as drivers or ‘champions’ giving them responsibilities for innovations related closely to their interest or areas of ‘specialization’. Through understanding the personalities and group dynamics of staff, managers described working with ‘gatekeepers’ to influence attitudes and behaviour for innovation adoption.

Managers linked their ability to harness staff dedication to the provision of teambuilding. They described a strong sense of teamwork and camaraderie with staff supporting each other and coming up with their own solutions to manage the challenges of high patient burden. In the absence of funds for teambuilding activities, many described initiatives taken by staff or management to organize social events such as shared meals or excursions outside working hours.

*I try to understand people's personality… identify the ring-leaders, the strong people, the people with influence… and then try to work with those people* (Participant 7).

To address some of the health system barriers to innovation adoption, most managers tried to use a participatory management style that encouraged staff involvement from initial planning stages. Further, some managers identified successful innovations that had come from the ‘floor’ led by frontline staff (e.g. introduction of ‘chronic clubs’ for patients taking antiretroviral therapy). Despite the value of staff inputs, most reported their role necessitated autocratic decision-making at times. In addition to staff input, many managers emphasized the importance of community input at the initial stages of new service planning to ensure innovations are responsive to the health needs of the community.

*Sometimes I think stats can be misinterpreted….so to plan a service according to stats is not the right way, for me, to go. Rather hear the need of the clinic and get the community involved and hear what their need is then plan a service* (Participant 19).

The ability to drive a process of building buy-in and motivating staff to move through their resistance was underscored by most participants. According to managers, building commitment to change involved providing adequate and timely information about the innovation, consulting staff before implementation and engendering a sense of competence and maintaining staff morale. Several managers expressed their role as one of understanding and ‘internalizing’ the innovation as presented from the health district, and then ‘packaging’ the information for comprehension by facility staff. Presenting innovations as tools for working ‘smarter’ was suggested as a strategy for obtaining staff support for change. Managers recognized the need for them to be available for ongoing discussions and questions from staff, and for them to allow sufficient time for these questions to emerge before implementing an innovation.

*And it took a lot of ground work and a lot of work to go back and say OK guys let’s not look at this as something in addition to your work but let us use this as a tool for how we can do our work better day to day… And you need to be available for questions, for support as well* (Participant 21).

Related to this, several managers recommended building in a pilot phase into the introduction of service innovations. Several managers described how innovations are often rapidly rolled out across districts without proper piloting or planning. They suggested an alternative approach involving piloting the innovation in one sub-district before a phased approach to scaling up implementation. They thought this would allow potential challenges to be identified and addressed thereby preventing the development of resistance. Managers recommended that due consideration should be given for when health innovations are introduced. They identified several time pressure points (such as annual audits, harvest time for farming regions and the annual surge in paediatric service demand due to diarrhoeal disease) that could negatively impact on innovation adoption. A commonly reported strategy for promoting uptake of innovations was working with unions and shop stewards. According to managers, the health workforce has strong unions, whose buy-in was crucial for service changes and managers described generally positive relationships with unions.

*They [facilities] each have their own ways of doing things… so, proper planning is always key to implementation. What happens is that we get a new protocol and …we go straight to implementation. Testing doesn’t mean you are not going to do it. Testing is just to find the best way to do it. I see it that way, but there’s, there’s no time for that* (Participant 2).

A subgroup of managers identified incorporating an ongoing review process into implementation planning as key to innovation adoption. They described providing ongoing support and guidance, ‘hand holding’, debriefing and problem-solving in overseeing the implementation process. Practical approaches included regular informal ‘check-ins’, setting aside time at regular intervals to reflect as a team on successes and challenges encountered, and learning opportunities to improve implementation.

*… from my experience, we had to keep coming back to the drawing board, telling each other this went well, this didn’t go so well… and revisit and reflect what was good and what was not so good* (Participant 23).

## Discussion

This study describes healthcare managers’ experiences of implementing health innovations in relation to complex interventions such as Project MIND and nationally initiated quality improvement initiatives and protocol changes. Although these managers oversee the adoption of health innovations, their experiences in this regard have rarely been investigated in South Africa. The need for training and professional development for non-physician health workers in the African context to align with disease burden and health system settings has been asserted (Couper *et al.*, 2018). Similar capacity building developments are required for managers. The perception of managers in this study that ‘doing more with less’ is unfeasible indicates the need to bolster skills of managers for functioning in their demanding work environment to support the implementation of health innovations (Gilson, 2013; Gilson *et al.*, 2014a, 2017). The behaviours and capabilities of managers described in this study appear to help address staff resistance to change. Whilst developed in the context of integration of mental health services into the chronic care platform, findings are likely to be relevant to PHC service delivery in general.

This study identified several individual- and health system-related constraints that managers experience when trying to implement health innovations. The complex milieu identified by managers echoes findings from previous South African studies highlighting lack of resources, inadequate staff skills and intricate interpersonal and hierarchical relationships (Gilson, 2003; Gilson *et al.*, 2005, 2014a; Daire and Gilson, 2014; Scott and Gilson, 2017). Staff shortages, increasing patient burden and the effect on workload, as reported in this study, are enduring challenges in LMIC PHC settings (Bradley *et al.*, 2013; Gilson *et al.*, 2017). These are compounded by ‘change fatigue’ (Aarons et al. 2014a; [Bibr czz017-B46][Bibr czz017-B40]) from multiple policy directives.

A major health system constraint identified in this study was the poor communication around introducing new innovations to frontline staff. This led to staff perceiving any proposed innovation simply as extra work. Resistance to change and lack of shared vision for progress on health outcomes amongst frontline staff was commonly described. Addressing the root cause of this resistance, namely workload, is essential, rather than attempting to force heath staff and the health system to cope with this challenge indefinitely (Munyewende *et al.*, 2014; Barasa *et al.*, 2017). However, managers also noted that frontline staff’s lack of understanding of the importance of health innovation to service delivery also contributed to this resistance. This was grounded in their lack of training in a public health approach to service delivery. Addressing this barrier necessitates clinician, and particularly nurse, training in South Africa evolving to include basic public health competencies. In the meantime, managers try to ‘package’ and ‘sell’ innovations to frontline staff using a public health approach that promotes the potential benefits (to patients and staff) of health innovations that improve disease outcomes at the population level (Gilson *et al.*, 2014a,b). Apart from their role as ‘innovation promoter’, managers also recognized their function as role models for promoting positive attitudes to work and to health innovations (Aarons *et al.*, 2014b). Capabilities in this area may be strengthened by building manager’s knowledge of relevant behaviour change, organizational, motivational and management theories and practices. In this way, managers can influence the organizational climate to engender constructive shared norms and values (Gilson *et al.*, 2014a; Scott *et al.*, 2014; Okello and Gilson, 2015), and create a learning environment conducive to change (Caldwell *et al.*, 2008).

Findings from the study also indicate the need for a streamlined process for planning, introducing, piloting, monitoring and providing feedback on innovations, enabling cycles of implementation learning (Berwick, 1998) with minimal administrative burden. Several aspects of planning emerged in this study as particularly relevant for innovation adoption. First, managers noted that staff have varying interests and strengths and these staff can be leveraged to promote the adoption of a health innovation within their interest area. Other studies have also noted that the presence of a ‘champion’ can be key to integration of mental health services into primary care (Chang *et al.*, 2013) and for other innovations. While managers described an organic process of getting to know staff interests, a more defined mapping process could uncover additional interests and aid planning. Managers also identified the utility of working with opinion leaders within the facility such as staff with strong personalities and union representatives, to champion change. Managers can also role model creative thinking, problem-solving, teamwork and explicit goal setting, and positive working relationships (Longo, 2007; Becan *et al.*, 2012) as building blocks towards innovation adoption.

Related to this, the hierarchical structure of the healthcare system also acted as a constraint by preventing both managers, frontline staff and service users from contributing to decision-making processes around the introduction of health innovations, which increased resistance to change. Most managers tried to address this by having a participatory management style to ensure the voices of frontline staff and community members are incorporated into planning for and monitoring of service delivery. Managers did, however, experience substantial limitations to operationalizing this approach due to the health system hierarchy and the urgency attached to implementing policy directives. To create facility environments that facilitate innovation adoption, it may be beneficial to support managers to deliver on this participatory management approach, e.g. through linking new managers with more experienced managers in a peer-learning approach (McConnell, 2002; Eyles *et al.*, 2015; Mbau and Gilson, 2018). Reinforcing community engagement platforms is indicated as well as development of a structure for bottom-up communication specifically around innovation adoption.

In this challenging work environment, managers highlighted the value of motivating frontline staff through teambuilding, support and providing recognition and rewards where possible. Trusting relationships between frontline staff and management appeared to exert a strong influence on staff motivation, retention in service and quality of care (Okello and Gilson, 2015) and may also influence the adoption of innovations (Scott *et al.*, 2012). Understanding and explicitly building trusting relationships with staff may enable managers to pave the way for innovation adoption. Strong relationships within the health system, particularly with other managers and across partner organizations can contribute to building these skills in individual managers (Gilson *et al.*, 2017).

**Figure 1:**
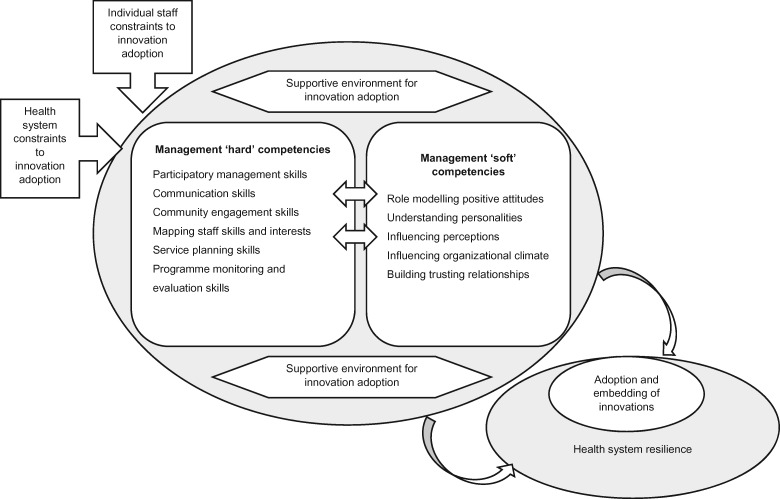
Framework of managerial competencies supporting innovation adoption and health system resilience.

Based on the emergent behaviours in this study, we developed a framework outlining managerial competencies that, when harnessed, could mitigate the impact of these constraints on the implementation of innovations (Figure 1). More specifically, these emergent behaviours appear to comprise a set of ‘hard’ or technical and ‘soft’ or non-technical management competencies which, when present, could moderate the impact of individual and system constraints on facility resilience and sustained implementation of an innovation. Developing and enhancing these competencies could form the basis of initiatives to strengthen managerial capacity for innovation adoption within PHC facilities in South Africa and other LMIC settings with similar health system challenges. We anticipate that many facility managers will be open to these initiatives, given that the majority described limitations in their skills for promoting innovation adoption and limited time for management tasks in general. In addition, supportive tools (such as planning documents, presentation templates and mapping tools) can be developed to aid the application and objective assessment of many of these competencies. The framework appears to have face validity as it aligns well with factors known to influence broader health system resilience including: preparedness and planning; leadership practices; organizational culture; and social networks and collaboration (Barasa *et al.*, 2017), with developments in high-income country healthcare settings building leadership and organizational climate for adoption of innovations (Aarons *et al.*, 2011, 2014a, 2015), and with synthesis of evidence for factors increasing likelihood for innovation adoption (e.g. positive attitudes to change in individual staff, individual skills and experience, supportive social environment; Wisdom *et al.*, 2014; Kelly *et al.*, 2017). However, the impact of efforts to build these managerial competencies in creating an environment supportive of change in South African PHC settings must still be established. In addition, managers in this study frequently reported considerable mental health challenges and stress related to their role, not only in relation to innovation adoption (manuscript in preparation). This suggests the need to incorporate mental health promotion and building resilience in the intervention developed.

Several limitations are acknowledged**.** There may be a selection bias excluding experiences of those who did not agree to be interviewed. A social desirability response may have been present due to managers having had previous interaction with the researchers. The experiences of managers are likely to be defined by the specific resourcing constraints of their facilities. These may vary both within the province and across other provinces, especially as there is provincially devolved responsibility for health policy implementation in South Africa. There may be limits of the relevance of these findings to other health services in South Africa and other LMICs.

## Conclusion

The question of whether it is feasible to ‘do more with less’ is fundamental to health systems strengthening and resilience in South Africa and globally. Closing the evidence to practice gap in which health innovations fail to be routinely delivered in PHC services may be one strategy to make this feasible. Many innovations fall away after an initial period of implementation if they are not embedded into the services’ standard practice. Managers have a vital role in this embedding process. This study has presented a framework for building capacity of managers for supporting innovation adoption leveraging their position as role models and influencers on organizational context and relationships. There are large gains to be made from investments to support managers in this way, given the range of innovations being introduced to PHC services. Future research should focus on co-production of a capacity building programme with researchers and health planners, and subsequent evaluation of managers’ progress towards building the managerial competencies specified in the framework.

## Ethics approval

Ethical approval was granted by the South African Medical Research Council Ethics Committee (EC004-2/2015). Written informed consent was obtained from participants prior to interviews.

## Author contributions

CBS and BM conceptualized the study. CBS conducted interviews and analysis and prepared the manuscript first draft. PPW was second coder. JK and HM provided health systems and public health specialist consultation and facilitated engagement managers. All authors edited and agreed on the final manuscript.

## Supplementary Material

Online Appendix 1
