# Supplementary material for: ‘Doing more with less’: a qualitative investigation of perceptions of South African health service managers on implementation of health innovations
Source: Health Policy Plan. 2019 Mar 12;34(2):132–40. doi: 10.1093/heapol/czz017 (PMC6481285; doi:10.1093/heapol/czz017)
Supplement: Online Appendix 1 [file czz017_online_appendix_1.docx]

**Strengthening South Africa's health system through integrating treatment for mental illness into chronic disease care (Project MIND)**

**Interviews with Service Providers**

**Interviewer to read:** Thank you for taking some time out of your busy schedule and agreeing to this interview. First, we would like to thank you for your support of project MIND. The purpose of this interview is to understand your perspective on the introduction of new programmes or services to your facility/district. This may help us to improve the implementation of project MIND and other health programmes. Please remember whatever you tell us in this interview will remain confidential and no names will be used in any reports.

1. What is your experience with introducing new programmes or services such as project MIND into your facility?

Probes:

1. *What is your role in implementing new programmes/services?*
2. *What makes introducing new programmes/services challenging?*
3. Can you give an example of a new programme/service that was introduced recently? (other than project MIND)

Probes:

1. *What were the things that helped it to be successfully introduced?*
2. *What factors make it more likely that the programme/service will be continued in the long term?*
3. If you think of chronic disease care, with all the possible new services that could be introduced, how are decisions made about what new programmes/services to introduce?

Probes:

*1. What is the role of the substructure?*

*2. What is the role of provincial Department of Health?*

*3. Do facilities themselves have a role?*

1. What can you do if you want to introduce a new service based on a need you recognise?

Probe:

1. *Can you give an example? Have you introduced something new based on patients’ needs, or staff feedback?*
2. What support do you get when new programmes/services are being introduced?

Probe:

1. *Staff training? Other?*
2. *What could be changed or improved?*
3. *What works well currently?*
4. What do you need to help you implement new services/programmes?

Probe:

1.Management mentoring/coaching?

2. Staff training? Other?

1. In your opinion, why are some new programmes/services more likely to be taken up and adopted by facilities, and others are not?

Probe:

1. *If you could change anything about the way new programmes/services are introduced, what would it be?*

**Interviewer to read:** Now let us talk about new services or programmes in relation specifically to integrated chronic disease care.

1. What do staff need guidance or support on to be able to provide integrated chronic disease care?

Probe:

1. *Using evidence-based practices, understanding patients’ needs?*
2. Does your facility have a clear plan for providing integrated chronic care?

Probes:

1. *What are staff roles?*
2. *What training is required?*
3. In your opinion, how is the communication between staff? And between managers and staff?

Probe:

1. *Formal communication, informal communication*
2. *What is your preferred communication style?*
3. As a manager, how do you encourage staff/facilities to take a positive and enthusiastic approach to new services/programmes?

Probe:

1. *How do you motivate people to take on what could be seen as ‘extra work’?*
2. *Leadership style – autocratic? Democratic?*
3. How would you describe the relationship between staff members?

Probe:

1. *Does this affect the facility’s ability to take on new programmes/services? Positively? Negatively?*
2. Where do pressures to change come from for your facility, e.g. pressures to introduce a new programme or service?

Probe*:*

1. *Staff, patients, substructure, provincial?*
2. *Are you able to make changes if you think there is a better way of doing things? Can you give an example?*
3. To what extent does staff turnover challenge the provision of services in your facility?

Probes:

1. *How do you do to try to retain staff?*
2. *What are the main reasons staff leave?*
3. *Do you have a way of getting ideas and feedback from staff?*
4. Can you describe the main challenges you face due to resource constraints?

Probes:

1. *How does this impact on the introduction of new programmes or services?*
2. *Do you feel you have the power to make changes?*
3. What is the situation with workload in your facility/district?

Probes:

1. *Does this impact introduction of new programmes/services?*
2. *Is workload shared equally as far as possible?*
3. In your opinion do your staff work together well as a team?

Probes:

1. *Do they have shared goals?*
2. *Do they contribute openly in meetings and other situations?*
3. *Are they generally accepting of new programmes?*
4. Is there anything you would like to add to what we have discussed today?

***Read:*** *These are all the questions I have for you today. You have provided us with a lot of useful information in this short amount of time. Thanks for your time—we appreciate your help.*
